# Pharmacokinetics and pharmacodynamics of intra-articular isoflupredone following administration to horses with lipopolysaccharide-induced synovitis

**DOI:** 10.1186/s12917-022-03537-5

**Published:** 2022-12-13

**Authors:** Heather K. Knych, Daniel Weiner, Linda Harrison, Daniel S. McKemie

**Affiliations:** 1 K.L. Maddy Equine Analytical Pharmacology Laboratory, School of Veterinary Medicine, University of California, 620 West Health Science Drive, Davis, CA 95616 USA; 2 Department of Molecular Biosciences, School of Veterinary Medicine, University of California, Davis, CA USA; 3 Pharmacometrics Consultant, Chapel Hill, NC USA; 4 Willow Oak Equine, Woodland, CA USA

**Keywords:** Horse, Isoflupredone, Corticosteroid, Pharmacokinetics, Pharmacodynamics, Inflammation

## Abstract

**Background:**

Intra-articular corticosteroids, such as isoflupredone acetate, are commonly used in the treatment of joint inflammation, especially in performance horses. Following administration in a non-inflamed joints blood concentrations of isoflupredone were low and detectable for only a short period of time post-administration compared to synovial fluid concentrations. For some drugs, inflammation can affect pharmacokinetics, therefore, the goal of the current study was to describe the pharmacokinetics of isoflupredone acetate following intra-articular administration using a model of acute synovitis. Secondarily, pharmacodynamic effects, including effects on joint circumference, joint flexion, and lameness following intra-articular administration of isoflupredone acetate in the experimental model were described.

**Methods:**

Sixteen horses received a single intra-articular dose of 8 mg of isoflupredone acetate or saline 12 h post-administration of lipopolysaccharide. Blood and urine samples were collected up to 72 h and synovial fluid for 28 days post-administration, drug concentrations determined by liquid chromatography- mass spectrometry and pharmacokinetic analysis performed. Joint circumference, maximum angle of pain free joint flexion and lameness were evaluated prior to and post-treatment.

**Results:**

The maximum isoflupredone plasma concentration was 2.45 ± 0.61 ng/mL at 2.5 ± 0.75 h and concentrations were less than the limit of quantitation by 72 h. Isoflupredone was below detectable concentrations in urine by 72 h post-administration in all horses and no longer detectable in synovial fluid by 96 h post-administration. Joint circumference was significantly decreased in the isoflupredone treatment group compared to the saline group at 24 and 48 h post drug administration**.** Pain free joint flexion was significantly different between the saline and isoflupredone treatment groups on day 4 post-treatment.

**Conclusions:**

Synovial fluid concentrations and maximum plasma concentrations of isoflupredone differed slightly between the current study and a previous one describing administration into a non-inflamed joint, however, the detection time of isoflupredone in blood was comparable. Effects of isoflupredone on joint circumference and degree of pain free joint flexion suggest a short duration of effect with respect to alleviation of lipopolysaccharide induced synovitis, however, results of this study support future studies of the anti-inflammatory effects of intra-articular isoflupredone acetate.

**Supplementary Information:**

The online version contains supplementary material available at 10.1186/s12917-022-03537-5.

## Background

Intra-articular corticosteroids are commonly used in the treatment of joint inflammation, especially in performance horses. Isoflupredone acetate is one of four corticosteroids labeled for intra-articular administration in this species by the Food and Drug Administration. Blood and synovial fluid concentrations as well as the pharmacokinetics of this drug in horses have been reported previously following intra-articular administration in healthy joints [1]. Similar to what has been reported for other intra-articular corticosteroids, blood concentrations were low and detectable for only a short period of time post-administration relative to synovial fluid concentrations.

As a relatively small molecular weight molecule, isoflupredone (after undergoing hydrolysis of the acetate ester) is cleared relatively quickly from the joint, presumably via capillaries and to a lesser extent via lymphatic drainage. In cases of acute joint inflammation, blood flow and capillary fenestrations have been shown to increase which in turn can increase vascular permeability [2]. These alterations can then lead to an increased rate of elimination of drugs from the synovial fluid, especially if administered via the intra-articular route of administration.

Several well-established experimental models have been used to study the effects of anti-inflammatory drugs on inflammation and pain. One such model involves intra-articular administration of lipopolysaccharide (LPS). This is a well characterized experimental model of acute synovitis and has been used in horses [3] to describe the pharmacokinetics and pharmacodynamics of opioids [4], local anesthetics [5] and other corticosteroids, including triamcinolone acetate [5] and dexamethasone sodium phosphate,[6], in the presence of synovial inflammation in horses. To the best of the authors’ knowledge, there are no reports describing the intra-articular pharmacokinetics of isoflupredone in an inflamed joint. To that end, in the current study we sought to describe synovial fluid, plasma and urine concentrations and the pharmacokinetics of isoflupredone following intra-articular administration using a model of acute synovitis. A secondary objective was to describe the pharmacodynamic effects, including effects on joint circumference, joint flexion, and lameness of intra-articular isoflupredone acetate using the LPS model.

## Results

The concentration–response relationships (relationship between calibrators and the LC–MS/MS instrument response) for isoflupredone in blood and urine were linear and had correlation coefficients of 0.99 or better. The precision (reported as percent relative standard deviation) and accuracy (reported as percent nominal concentration) were determined by assaying quality control samples in replicates (*n* = 6). Accuracy and precision for isoflupredone in blood, urine and synovial fluid were considered acceptable based on the Food and Drug Administration’s guidelines for Bioanalytical Method Development (Table 1) [7]. The limit of quantitation (LOQ) was the lowest calibrator that could be measured with acceptable precision and accuracy and the limit of detection (LOD) was established based on the lowest calibrator with a 3:1 signal to noise ratio. The LOQ was 0.05 ng/mL and a the LOD was approximately 0.04 ng/mL for isoflupredone in plasma, urine and synovial fluid.

**Table 1:** Accuracy and Precision Values for LC–MS/MS analysis of isoflupredone in equine plasma, urine and synovial fluid

	Concentration (ng/mL)	Accuracy (% nominal conc)	Precision (% relative SD)
Plasma	0.15	96.0	12.0
	2.0	108	10.0
	9.0	115	10.0
	0.15	96.0	20.0
Urine	0.3	114	8.0
	2.0	101	10.0
	9.0	88.0	6.0
	3.0	104	9.0
Synovial Fluid	750.0	104	8.0
	4000.0	95.0	7.0

Isoflupredone concentrations were below the LOQ of the analytical assay in plasma by 72 h post-administration (Table 2). Isoflupredone concentrations in the joint were above the LOD of the analytical assay in the right antebrachiocarpal joint at 72 h post-administration and no longer detectable by 96 h (Table 2). Isoflupredone was detected in the right middle carpal joint in 5 of 8 horses at 24 h and was below detection limits by 48 h in all horses (Table 2). Isoflupredone was not detected at any time in synovial fluid collected from the left antebrachiocarpal or middle carpal joints.

**Table 2:** Mean (± SD) plasma and synovial fluid isoflupredone concentrations following a single intra-articular administration of 8 mg of isoflupredone acetate (Predef**®** 2X) in the right antebrachiocarpal joint 12 h post intra-articular LPS administration to exercised Thoroughbred horses (*n* = 8)

	**[Isoflupredone] (ng/mL)**				
Time	Plasma	Right Antebrachiocarpal Joint	Right Middle Carpal Joint	Left Antebrachiocarpal Joint	Left Middle Carpal Joint
Baseline	ND	ND	ND	ND	ND
0.25 h	0.37 ± 0.15	–-	–-	–-	–-
0.5 h	0.66 ± 0.29	–-	–-	–-	–-
0.75 h	0.79 ± 0.32	–-	–-	–-	–-
1.0 h	0.81 ± 0.40	–-	–-	–-	–-
1.5 h	0.87 ± 0.34	–-	–-	–-	–-
2.0 h	1.16 ± 0.44	–-	–-	–-	–-
2.5 h	1.25 ± 0.48	–-	–-	–-	–-
3.0 h	1.46 ± 0.52	–-	–-	–-	–-
4.0 h	1.59 ± 0.47	–-	–-	–-	–-
6.0 h	1.59 ± 0.35	–-	–-	–-	–-
8.0 h	1.29 ± 0.36	–-	–-	–-	–-
12.0 h	1.07 ± 0.26	–-	–-	–-	–-
18.0 h	0.49 ± 0.13	–-	–-	–-	–-
24 h	0.19 ± 0.04	828.9 ± 608.4	0.97 ± 0.77	–-	–-
36 h	0.09 ± 0.03	–-	–-	–-	–-
48 h	< LOQ	14.2 ± 10.8	ND	–-	–-
72 h	< LOQ	0.68 ± 0.20	ND	–-	–-
96 h	–-	ND	ND	ND	ND
120 h	–-	ND	ND	–-	–-
Day 7	–-	ND	ND	–-	–-
Day 9	–-	ND	ND	ND	ND
Day 10	–-	ND	ND	–-	–-
Day 14	–-	ND	ND	–-	–-
Day 21	–-	ND	ND	–-	–-
Day 28	–-	ND	ND	ND	ND

ND, not detected; LOQ, limit of quantitation; –-, no sample collected

**Fig. 1:**
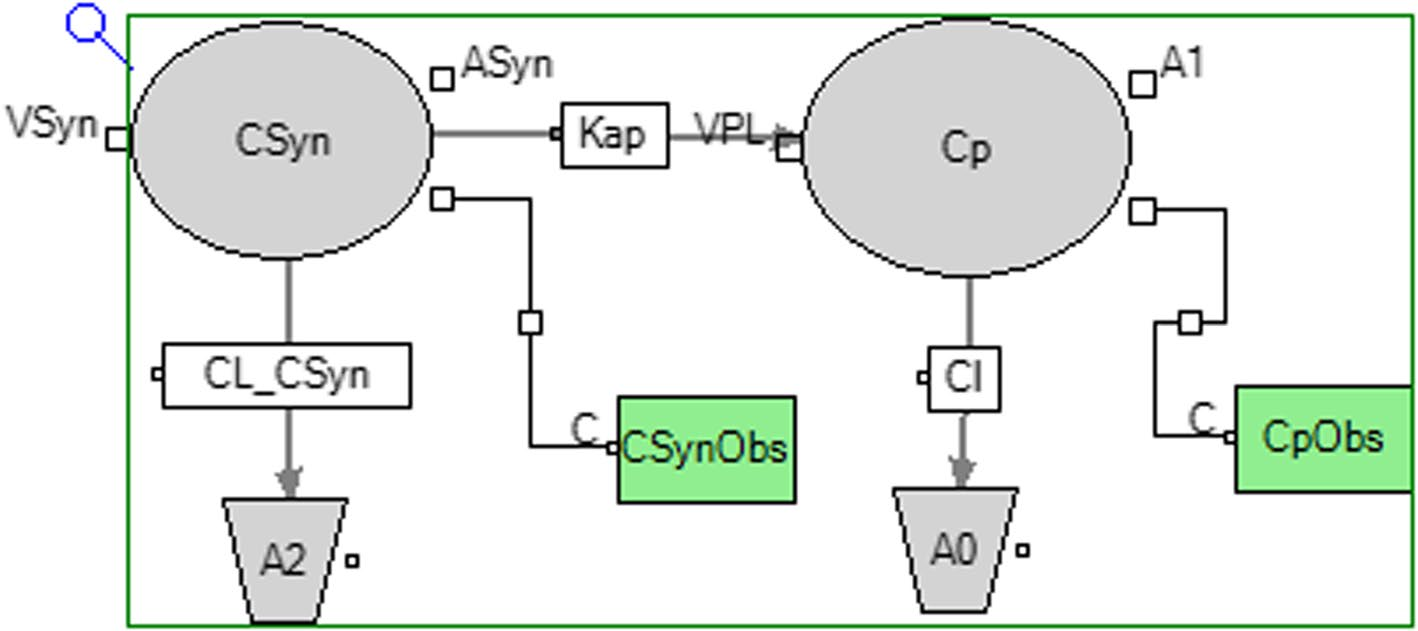
Isoflupredone synovial fluid and plasma disposition pharmacokinetic model. Parameters are defined in Table 3

**Fig. 2:**
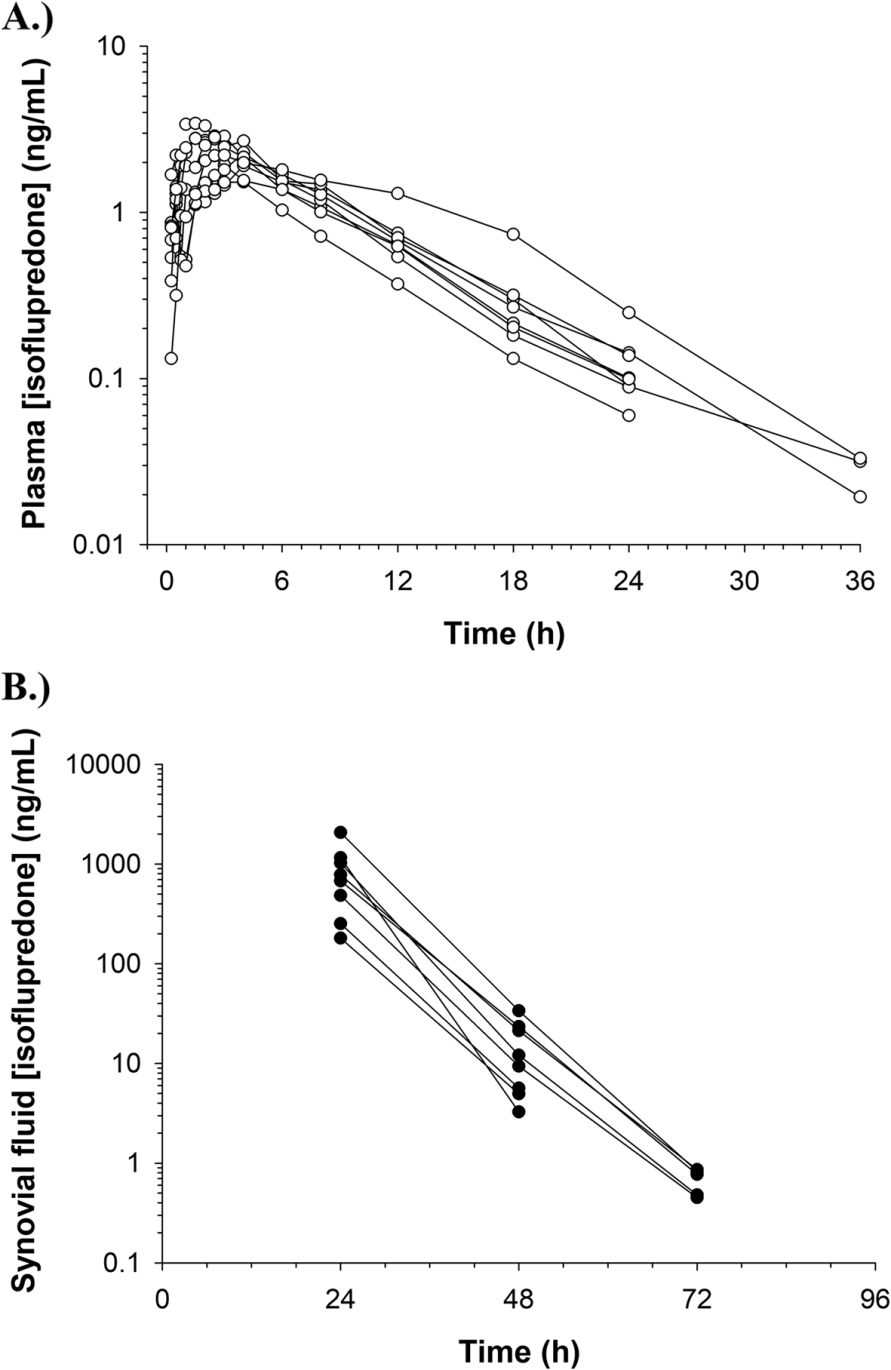
Isoflupredone **A** plasma and **B** synovial fluid concentration time curves for 8 exercised Thoroughbred horses following intra-articular administration of a single 8 mg dose in the right antebrachiocarpal joint following intra-articular LPS administration

**Fig. 3:**
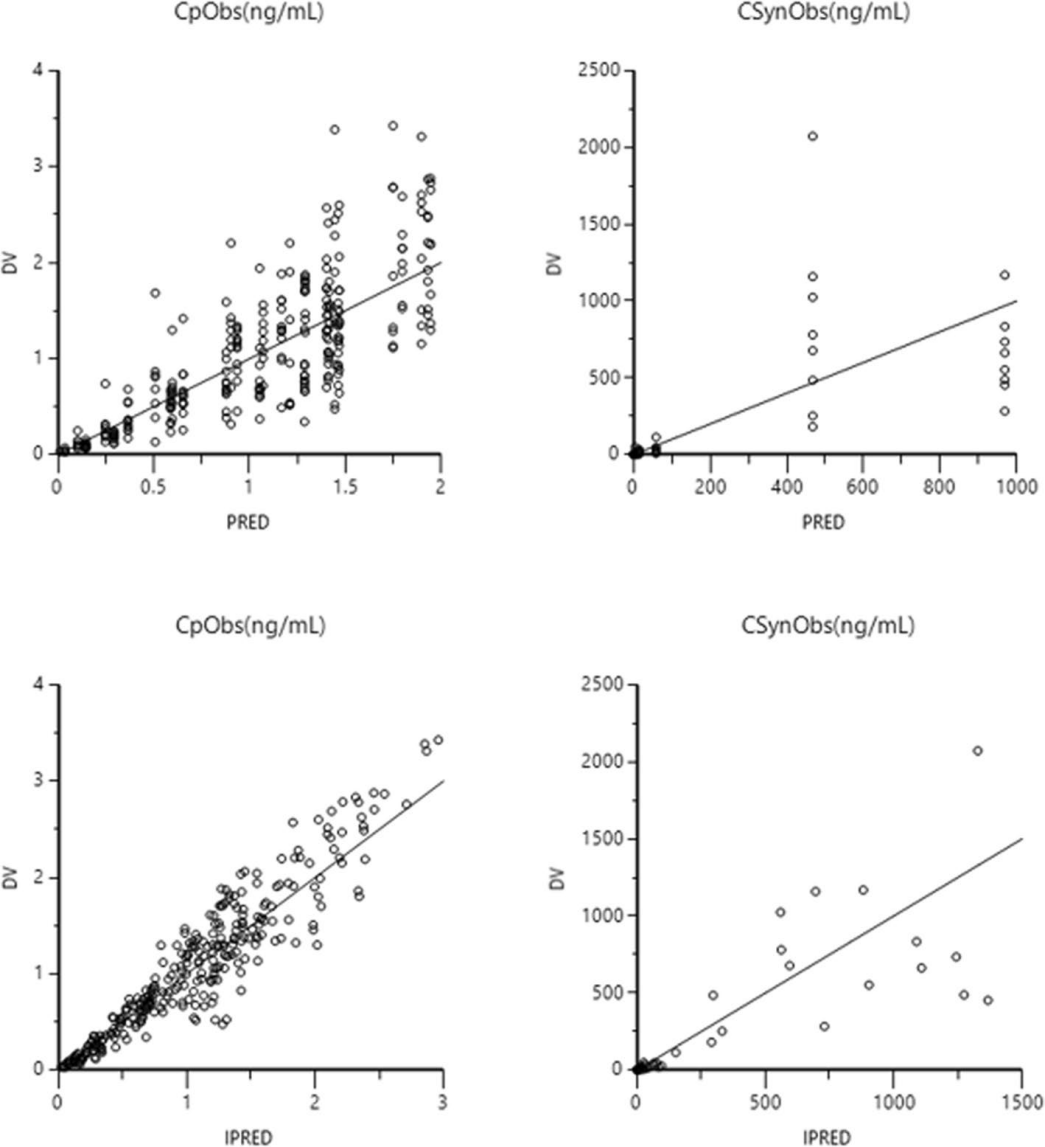
Diagnostic plots for fit of intra-articular isoflupredone concentration data using a1-compartment model with a multiplicative error. Upper plots are predicted (PRED) versus dependent variable (DV) for plasma and synovial fluid and lower plots are individual predicted (IPRED) versus dependent variable (DV) for plasma and synovial fluid

The isoflupredone plasma and synovial concentration time curves are depicted in Fig. 1. The maximum plasma concentration (C_max_ mean(range)) was 2.50 (1.67–3.43) ng/mL and T_max_ (mean(range)) was 2.50 (1.50–4.0) hours. Modeling of absorption of the drug from plasma back into the synovial fluid was attempted, but it was much lower in value (K_ap_ = 0.0001 1/h), such that the rate of absorption from synovial fluid to plasma could not be reliably estimated. Based on the estimated values of the parameters and their good precision and based on an examination of the diagnostic plot, the final pharmacokinetic model used a multiplicative residual error model for both the plasma and synovial fluid data. The final model and diagnostic plots are provided in Figs. 2 and 3. Pharmacokinetic parameters (estimate, coefficient of variation) for the joint fitting of the synovial fluid and plasma data are listed in Table 3. While modeling it was determined that administration of LPS had no effect on the clearance of isoflupredone from synovial fluid or plasma, but the rate of absorption of drug from synovial fluid to plasma (Kap) increased to 0.107 in the presence of LPS from a value of 0.077 when LPS was not administered. Kap was significantly (*p* < 0.05) different in the presence of LPS administration (0.107 vs. 0.077 1/hr). When LPS was not administered, the t_1/2_ of absorption was 9 h. The plasma elimination t_1/2_ was 0.9 h regardless of whether LPS was administered.

**Table 3:** Model typical values (tv) for isoflupredone following a single intra-articular administration of 8 mg of isoflupredone acetate (Predef**®** 2X**)** in the right antebrachiocarpal joint 12 h post intra-articular LPS administration, to exercised Thoroughbred horses (*n* = 8)

Parameter	Estimate	CV%
tvV_plasma_ (mL)	423,442.6	19.7
tvCl (ng*h/mL)	337,682.9	11.2
tvV_syn_ (mL)	557.5	24.3
tvKap (1/h)	0.077	10.7
tvCL_syn_ (ng*h/mL)	22.5	33.2
dKapdLPS1	0.333	38.5
stdev0	0.249	4.67
stdev1	0.584	22.0
HL_plasma_ (h)	0.869	18.1
Kap_HL (h)	9.03	10.7
Syn_HL (h)	17.1	19.8
Kap_LPS(1/h)	0.107	11.6
Kap_LPS_HL(h)	6.47	11.6

tvV_plasma_ denotes the value of the plasma volume of isoflupredone; tvCl the clearance of drug from plasma; tvVSyn the clearance of drug from synovial fluid; tvKap the rate of absorption of isoflupredone from the joint into the plasma in the absence of LPS; tvCLCSyn the clearance of drug from synovial fluid; dKapdLPS1 the effect of LPS on Kap; stdev0 the estimated residual standard deviation for plasma data; stdev1 the corresponding value for the synovial fluid data; HL_plasma_, the elimination half-life of drug from plasma; KapHL the half-life of absorption of the drug into plasma from synovial fluid in the absence of LPS; Syn_HL the elimination half-life of drug from synovial fluid; Kap_LPS the rate the rate of absorption of isoflupredone from the joint into the plasma during LPS; Kap_LPS_HL the half-life of absorption of the drug into plasma from synovial fluid during LPS administration

Isoflupredone was below detectable concentrations in urine by 72 h post-administration in all horses (Table 4). Based on the sparse sample collection (only one horse had a detectable amount in the urine at two time points), urine data were deemed not acceptable for PK modeling.

**Table 4:** Urine isoflupredone concentrations following a single intra-articular administration of 8 mg of isoflupredone acetate (Predef**®** 2X**)** in the right antebrachiocarpal joint 12 h post intra-articular LPS administration to exercised Thoroughbred horses (*n* = 8)

Time	Horse 1	Horse 2	Horse 3	Horse 4	Horse 5	Horse 6	Horse 7	Horse 8
	Isoflupredone Concentration (ng/mL)							
0 h	ND	ND	ND	ND	ND	ND	ND	ND
24 h	–-	6.40	ND	3.32	2.48	1.71	5.24	1.78
48 h			ND	< LOQ	< LOQ	< LOQ	0.28	< LOQ
72 h	ND	ND	ND	ND	ND	ND	ND	ND

ND, not detected; –-, no sample

**Fig. 4:**
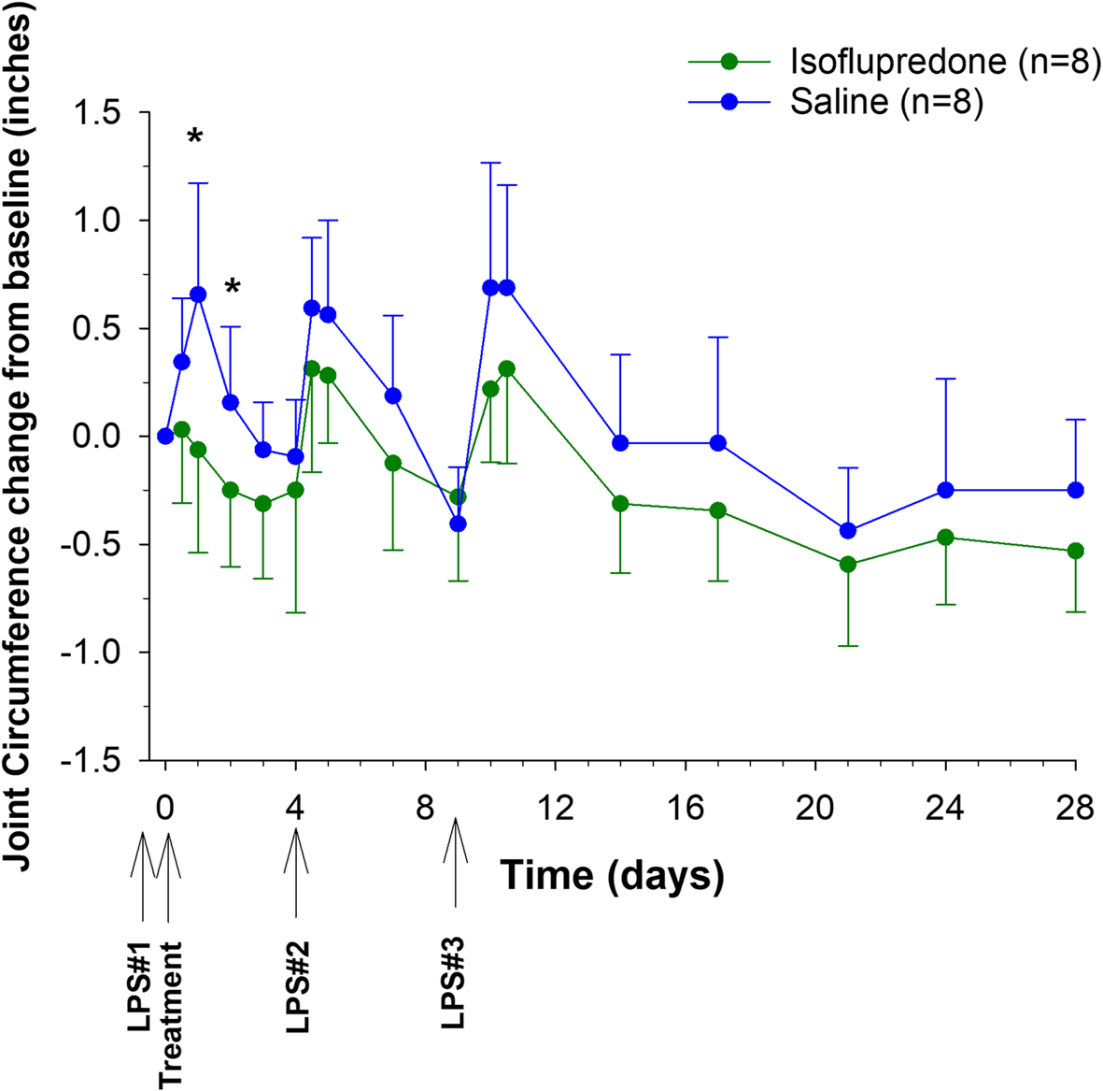
Joint circumference change from baseline for 16 exercised Thoroughbred horses following intra-articular administration of a single 8 mg dose of isoflupredone acetate or an equivalent volume of saline (4 mL) in the right antebrachiocarpal joint following intra-articular LPS administration. *, indicates a significant (*p* < 0.05) difference compared to baseline between isoflupredone and saline treatments

**Fig. 5:**
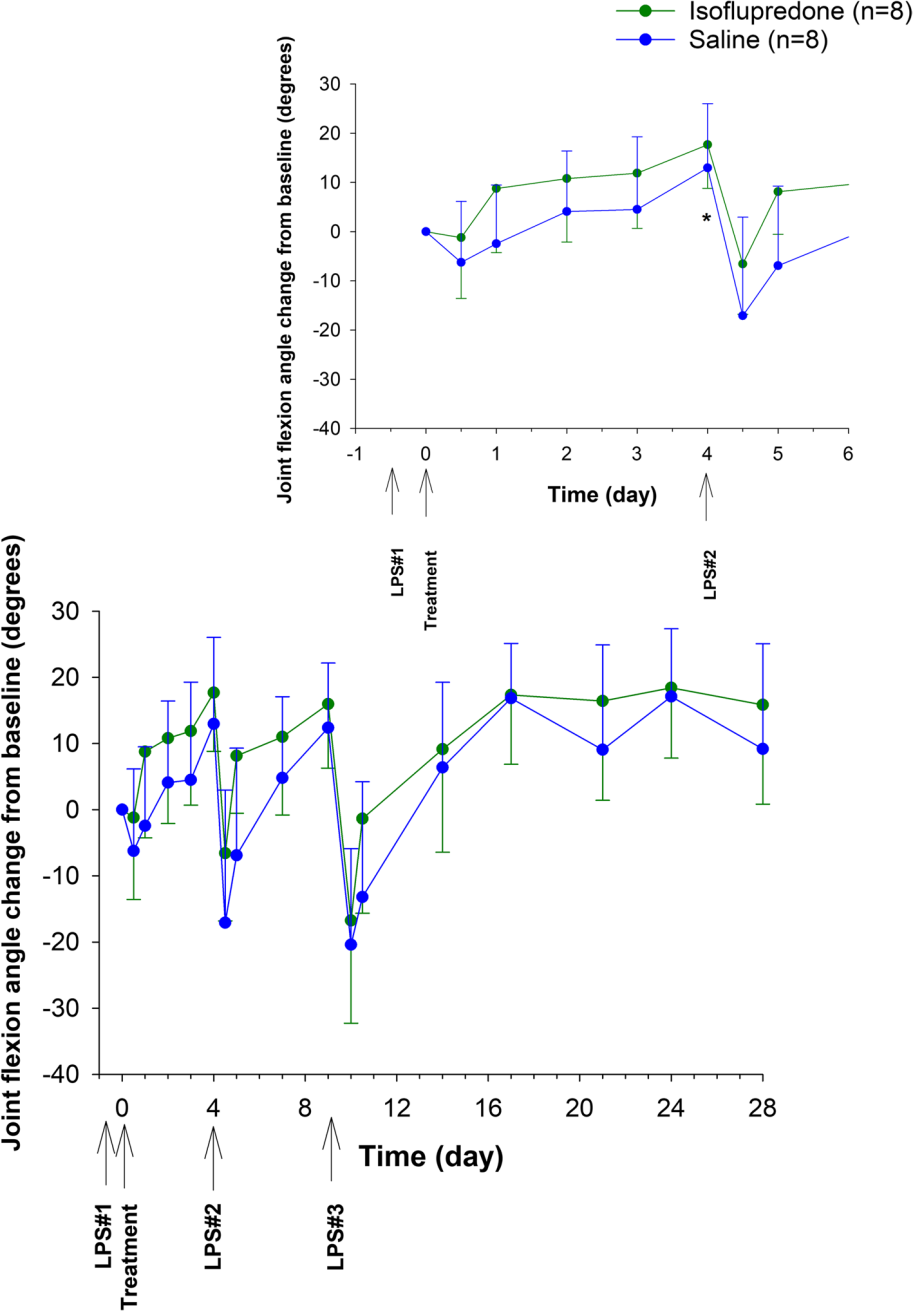
Maximum degree of pain free joint flexion (change from baseline) for 16 exercised Thoroughbred horses following intra-articular administration of a single 8 mg dose of isoflupredone acetate or an equivalent volume of saline (4 mL) in the right antebrachiocarpal joint following intra-articular LPS administration. The inset shows the first 6 days post the first LPS administration. *, indicates a significant (*p* < 0.05) difference compared to baseline between isoflupredone and saline treatments

The effect of treatment on joint circumference is depicted in Fig. 4. The change in circumference of the right antebrachiocarpal joint (post LPS_1_ administration compared to post-treatment with isoflupredone or saline) was significantly decreased in the isoflupredone treatment groups compared to the saline group at 24 and 48 h post drug administration. The change in the degree of pain free joint flexion compared to pretreatment values (Fig. 5) was significantly different between the saline and isoflupredone treatment groups on day 4 post-treatment.

**Fig. 6:**
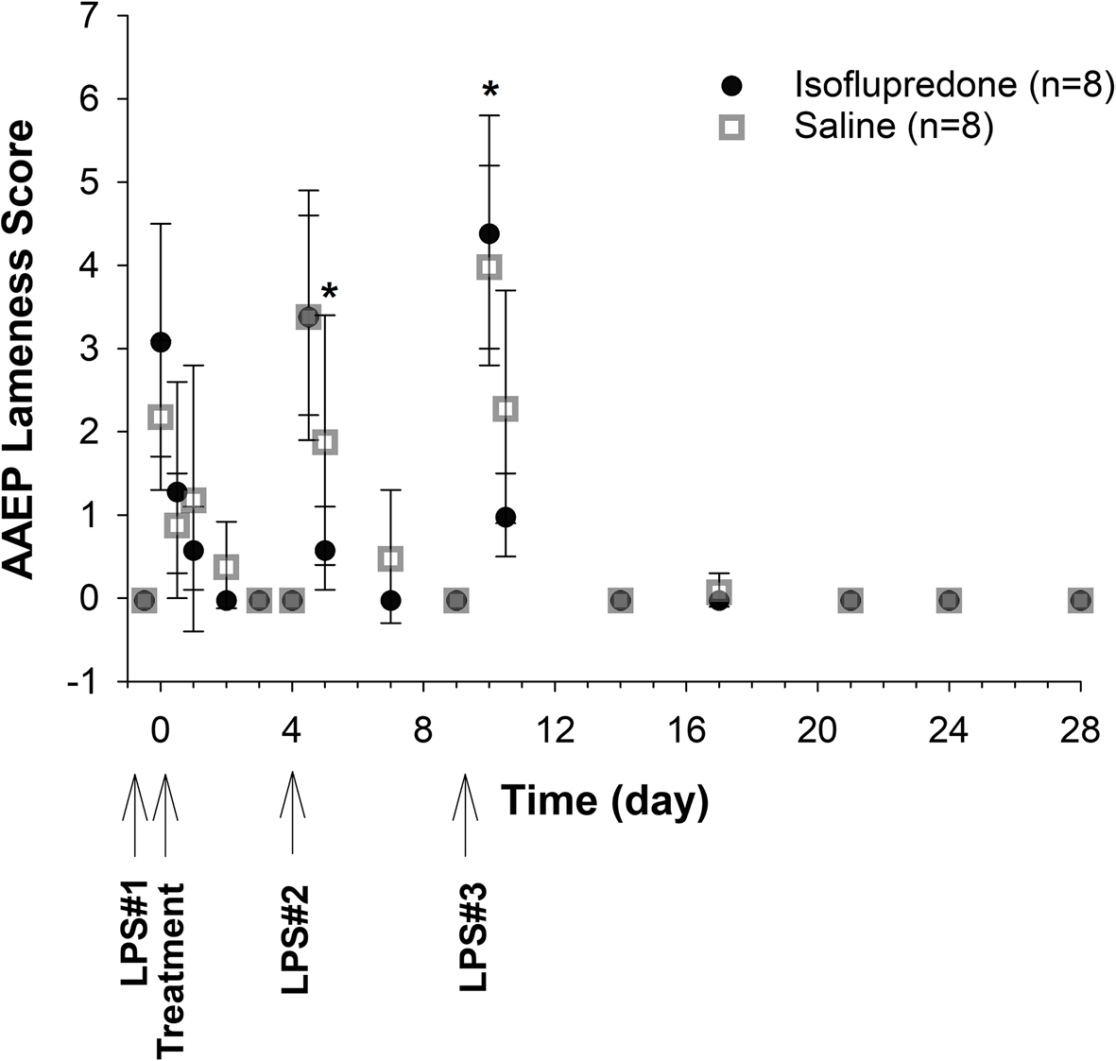
Lameness scores for 16 exercised Thoroughbred horses following intra-articular administration of a single 8 mg dose of isoflupredone acetate or an equivalent volume of saline (4 mL) in the right antebrachiocarpal joint following intra-articular LPS administration. *, indicates a significant (*p* < 0.05) difference compared to baseline between isoflupredone and saline treatments

All horses, in both the treated and control groups were sound (lameness score of 0) prior to LPS administration (Fig. 6). At 12 h post LPS administration (prior to treatment), lameness scores ranged from 1–4 for all horses in both dose groups. Lameness scores were reduced, relative to pre-treatment values and significantly different between the isoflupredone and saline groups on days 5 and 10 following treatment.

## Discussion

The goal of the current study was to describe the pharmacokinetics and anti-inflammatory effects of isoflupredone following intra-articular administration in a well-established model of acute synovitis. The pharmacokinetics of intra-articular isoflupredone in a non-inflamed joint have been reported [1] but to the best of the authors’ knowledge the potential effects of inflammation induced changes in the joint on drug disposition and a comparison of them to the non-inflamed state have not been described.

The maximum blood concentration (C_max_: 2.45 ng/mL) in the current study was slightly higher and T_max_ earlier (2.50 h) than reported following intra-articular administration in non-inflamed joints (C_max_:1.53 ng/mL and T_max_: 3.34 h) [1]. The detection time of isoflupredone in synovial fluid was also shorter (non-detectable by 4 days) compared to the previous study in which isoflupredone, administered at the same dose, was above the LOQ on day 4 in most horses studied and in one horse for between 14 and 21 days [1]. In addition to the joint of administration, drug was also detected in synovial fluid collected from the middle carpal joint. This is not unexpected based on the communication between the antebrachiocarpal and middle carpal joints and has been reported previously for isoflupredone acetate and other corticosteroids administered into the antebrachiocarpal joint in horses [1, 8, 9]. In agreement with a previous report describing the disposition of isoflupredone [1], the drug was not detected in the contralateral joints.

Although synovial fluid concentrations and plasma C_max_ and T_max_ differed slightly between the previous [1] and current study, the detection time of isoflupredone in blood was comparable with concentrations falling below the LOD by 48 h in both reports. In the current study, isoflupredone was no longer detected in urine samples in any of the horses at 72 h post-administration. This detection time is slightly shorter than that reported in the previous study by our group whereby isoflupredone was still detectable (< 0.05 ng/mL) in all 12 horses at 72 h and on day 7 in one horse [1].

Plasma data from the study previously published by our laboratory [1] was reanalyzed along with the data generated in this study. The increase in the number of animals allowed for an improved model fit and a more detailed model for both data sets. With the improved fit, isoflupredone concentrations from both studies were best described using a one-compartment model for each of the synovial fluid and plasma data. This is in contrast to the previous publication in which the plasma data were fit to a two-compartment model [1]. The rate of appearance of isoflupredone in plasma (K_ap_) following intra-articular administration to animals with acute synovitis was more rapid (0.107 1/h) compared to administration into a non-inflamed joint (0.077 1/h). While notably the number of animals studied was small and a limited number of samples collected, it is possible that the increased rate of exit from the joint and entry into the blood in the LPS group may be due to increases in vascular permeability associated with the induced synovitis. Once the drug reached the circulation, elimination from blood was rapid (0.9 h). The short plasma elimination half-life coupled with the relatively longer overall detection time suggest flip-flop kinetics, whereby the terminal portion of the concentration curve is more influenced by the rate of delivery (absorption) as opposed to elimination of the drug from the blood and hence the body. This behavior is not unexpected as the formulation administered in the current study was an ester form (acetate) and the intent of ester formulations is to prolong the residence time of the drug in the joint through slow release, the rate of which is dependent on enzyme hydrolysis.

The LPS model of inflammation has been used previously to describe the effects of corticosteroids on clinical indicators of inflammation [5, 6]. Similar to the protocol used by Kay and colleagues [5] when assessing the effects of intra-articular triamcinolone acetonide on LPS induced inflammation, in the current study, multiple intra-articular injections of LPS were administered to evaluate the reported prolonged anti-inflammatory effects of the isoflupredone ester formulation. As described previously [5], signs of inflammation, including an increase in joint circumference, decrease in joint flexion and an increase in lameness scores, were present within 12 h of each LPS administration in all horses studied. In the present study, although joint circumference decreased (relative to pre-treatment values) to a greater extent in the isoflupredone acetate treated group compared to the saline control group, this change was only significant at 24 and 48 h post-treatment, suggesting the duration of effect of isoflupredone on LPS induced synovitis is short-term. In previous studies describing the effects of intra-articular triamcinolone acetonide and dexamethasone 21-phosphate on experimentally induced synovitis, investigators reported a lack of significant change in joint circumference following intra-articular administration into a joint previously injected with LPS [5, 6]. In the dexamethasone study, the investigators suggested that joint circumference may not be a suitable marker for assessing drug induced anti-inflammatory effects in acute synovitis [6]. Similar to joint circumference, changes in joint flexion, relative to pre-treatment was greater in the isoflupredone acetate group compared to the saline group, but this increase in flexion was only significantly different between treatment groups at one time post-administration (4 days). Changes in joint circumference and flexion were not significantly different between treatment groups following administration of LPS_2_ and LPS_3_, further supporting the short duration of effect of isoflupredone on LPS induced synovitis. It is interesting to note that while changes were not significant, subjectively the isoflupredone acetate treated horses appear to recover more quickly than horses administered saline after LPS administrations.

Following the initial LPS administration, lameness scores increased in both treatment groups. Scores were reduced within 12 h of isoflupredone acetate administration until the second LPS administration, however, the same outcome was observed in the saline treated group. This finding suggests that the decrease in lameness was likely not related to treatment because this initial LPS administration was not able to maintain a visually discernable lameness. Except for two time points, statistical differences in lameness scores between treatment groups were not observed with subsequent administrations of LPS. Although notably, drug was administered 2 h post LPS administration, as opposed to 12 h in the current study, Ekstrand and colleagues similarly reported that intra-articular administration of dexamethasone phosphate did not decrease lameness scores when assessed using the same subjective scoring system used in the current study [6]. The investigators did, however, report significant decreases in lameness when utilizing the lameness locator tool which is considered to be a more objective assessment of lameness in horses.

Although consistent with previously published studies assessing the pharmacokinetics/pharmacodynamics of intra-articular corticosteroids in horses, one notable limitation in the current study is the relatively small sample size. It is also important to note that a relatively small number of synovial fluid samples were collected, with the first sample collection not occurring until 24 h post-administration. While this was deemed appropriate by investigators for animal welfare purposes, specifically to reduce the potential for infection and/or inflammation associated with the arthrocentesis procedure, the sparseness of the synovial fluid samples precluded fitting a combined pharmacokinetic/pharmacodynamic model to the data. Another notable discussion point is the choice of the inflammatory model used in the current study. Although the LPS model has been used in several studies designed to assess the effects of anti-inflammatory medications on joint inflammation, it is important to note that the inflammation is transient in nature and without intervention, inflammation typically resolves on its own within 36–48 h [3, 10]. While the benefit of this model is that effects are not permanent, the downside is that it allows for a very limited window in which to assess anti-inflammatory effects. Another potential limitation in the current study relates to the use of a fixed Kap when modeling the data. While the use of a fixed Kap resulted in good modeling of the data, because an anti-inflammatory presumably reduces vascular permeability over time, Kap may be time-dependent. To the best of the authors’ knowledge, there is no literature describing such an effect, but without a positive control and given the sparsity of the synovial fluid samples collected, this cannot be ruled out.

## Conclusions

In the study reported here, compared to intra-articular administration in a non-inflamed joint, the rate of appearance of isoflupredone in the plasma is more rapid and plasma C_max_ and T_max_ higher and earlier. With respect to future pharmacokinetic studies, data describing plasma concentrations following systemic administration as well as additional urine samples following IA administration would confirm the pharmacokinetic model utilized in the current study. Isoflupredone acetate increased pain free range of motion and transiently decreased lameness scores following a second and third LPS challenge. Based on the pharmacodynamic effects reported here, additional studies utilizing higher doses and/or additional models of inflammation are warranted to fully assess the anti-inflammatory effects of isoflupredone acetate following intra-articular administration.

## Methods

### Animals

Sixteen exercised healthy 4–7-year-old University owned Thoroughbred horses (8 mares and 8 geldings) were studied. The horses utilized for this study were part of an exercised research herd and were regularly exercised five days week utilizing a combination of workouts on a high speed-treadmill and Equineciser, following standard protocols established by our laboratory and described previously [1]. Due to repeated arthrocentesis, LPS administration and induced lameness, horses were not exercised during the study period.

Horses did not receive any medications for a minimum of four weeks prior to commencement of the study and were determined healthy by physical examination, complete blood count (CBC) and a serum biochemistry panel performed the day before the first LPS administration. Blood analyses were performed by the Clinical Diagnostic Laboratories of the William R. Pritchard Veterinary Medical Teaching Hospital of the University of California, Davis, using standard protocols. The study was conducted in accordance with the Institutional Animal Care and Use Committee of the University of California at Davis and according to ARRIVE guidelines.

### Experimental induction of inflammation/lameness

Synovial inflammation and forelimb lameness was induced in all sixteen horses using a previously described model of LPS induced synovitis [3, 5, 11]. Briefly, LPS (E.coli O55:B5; Sigma-Aldrich, St. Louis, MO) was prepared in a sterile manner, at a concentration of 100 ng/mL in Dulbecco PBS solution. The area surrounding the right antebrachiocarpal joint was clipped and prepared for injection and localized inflammation induced by sterile injection of 100 ng (1 mL) of LPS. Additional doses of LPS were administered in the same manner on days 4 and 9.

### Instrumentation and drug administration

Prior to drug administration, 8 horses were randomly assigned to the control (saline) group and 8 horses to the treatment (isoflupredone acetate) group, using a random number generator. The control group was included for pharmacodynamic assessments. Horses were treated with saline or drug 12 h post-administration (Day 0) of the first LPS dose. For horses receiving isoflupredone acetate, prior to drug administration, a 14-gauge catheter was placed in one external jugular vein for collection of blood samples.

For drug or saline administration, the area over the right antebrachiocarpal joint was scrubbed with chlorhexidine solution and 70% isopropyl alcohol, the joint flexed and a total dose of 8 mg of isoflupredone acetate (Predef 2X, Zoetis, Florham Park, NJ) or an equivalent volume of saline (control group) was administered aseptically into the joint. The dose chosen for this study was based upon the previous study conducted by our laboratory [1].

### Sample collection

For drug concentration determinations, blood samples were collected at time 0 and at 15, 30, and 45 min, and 1, 1.5, 2, 2.5, 3, 4, 6, 8, 12, 18, 24, 36, 48 and 72 h post isoflupredone acetate administration. Catheters were removed following collection of the 24-h sample and the remaining samples collected by direct venipuncture. Blood samples were collected into EDTA blood tubes, centrifuged at 3000 × g and plasma immediately transferred into storage cryovials and stored at -20◦C until analysis.

Synovial fluid samples were collected from the right antebrachiocarpal and middle carpal joints by aspiration with a sterile needle prior to LPS administration, immediately prior to drug administration and at 24, 48, 72, 96 and 120 h and on day 7, 9, 10, 14, 21 and 28-days post-administration. Samples were collected from the left leg prior to drug administration and on days 4, 9 and 28 post drug administration. Synovial fluid was stored at -20 °C until analysis for determination of drug concentrations.

Urine samples were collected from all horses via free catch for measurement of isoflupredone concentrations. Samples were collected on Day 0 (prior to drug administration) and at 24, 48 and 72 h post drug administration. All samples were stored at -20◦C until analyzed for determination of isoflupredone concentrations.

### Sample analysis (plasma/synovial fluid/urine drug concentrations)

The concentration of isoflupredone was measured in plasma, synovial fluid and urine by liquid chromatography-tandem mass spectrometry (LC–MS/MS) as described previously [1].

### Pharmacokinetic modeling of isoflupredone concentration data

Isoflupredone plasma concentration data from a previous study, in which 12 horses received a single intra-articular 8 mg dose of isoflupredone acetate (Predef 2X, Zoetis, Florham Park, NJ) into a non-inflamed joint [1] were pooled with the concentration data generated from the current study. This additional data allowed for determination of the most appropriate pharmacokinetic model for simultaneous modeling of the plasma and synovial fluid concentration data. Incorporation of this data set also allowed for a comparison of data generated in the two studies. Synovial fluid samples used in the analysis were those collected at 24, 48, 72 and 96 h post-administration.

For data generated in the current study (LPS model), a non-compartmental analysis (NCA) was performed on the plasma concentrations as an aid in determining initial estimates for subsequent model fitting. A number of models were fit to the data (Supplemental Data), including one and two compartments for each of the synovial fluid (Csyn) and plasma concentrations (Cp). In addition, the effect of LPS on the parameters was assessed via modeling LPS as a covariate. The final model that was employed used a single compartment for each of Csyn and Cp. The absorption of drug from the synovial fluid to plasma was modeled as irreversible, and separate rates of appearance of drug in plasma (Kap) values were determined for LPS vs non-LPS data. The final model used a clearance parameterization, and relevant half-lives of interest were derived from the clearances and volumes of distribution. The Csyn and Cp data for all horses were modeled simultaneously using a nonlinear mixed modeling approach with the Phoenix NLME software program (V8.3.5.340; Certara, Princeton, NJ).

Multiplicative residual error models were used for both Csyn and Cp data, and the effect of LPS on Kap was also modeled via an exponential function.

### Clinical examination

Pharmacodynamic assessments were determined immediately prior to LPS administration (-12 h prior to isoflupredone acetate or saline administration), time 0 (immediately prior to isoflupredone acetate or saline administration) and at 12, 24, 48, 72, 96 (immediately prior to LPS_2_) and 108 h and on days 5, 7, 9 (pre LPS_3_), 10, 10.5, 14, 17, 21, 24 and 28 post drug administration. All assessments were performed by an experienced board-certified equine surgeon (ACVS) blinded to treatment. For lameness evaluations, horses were required to walk and trot in a straight line and were scored according to guidelines established by the American Association of Equine Practitioners (AAEP) [12, 13]. The range of motion of the antebrachiocarpal joints was determined by passive flexion and the tendency to pull away. The joint was flexed and a goniometer positioned at the center of rotation of the joint was used to measure the maximal angle of flexion [5, 13]. Three consecutive measurements were taken, and the average reported. The degree of joint distension was measured using a tape to measure the circumference of the antebrachiocarpal joint.

### Statistical analysis

Changes from baseline (time 0) were computed for each of the horses and t-tests were performed separately for each time post dosing to determine if any of the changes from baseline for circumference, flexion and lameness scores were statistically significant between the saline and isoflupredone treatment groups. A level of statistical significance of 0.05 was used. These tests should be considered as descriptive in nature and no adjustment was made for the multiplicity of tests that were run.

## Supplementary Information

**Additional file 1.**

## Data Availability

The datasets used and/or analyzed during the current study are not publicly available for ethical reasons but are available from the corresponding author on reasonable request.

## Abbreviations

CBC: Complete blood count
C_max_: maximum concentration
Cp: Concentration in plasma
Csyn: Concentration in synovial fluid

## EDTA

Kap: Rate of appearance of drug in plasma
LC–MS/MS: Liquid chromatography mass spectrometry/mass spectrometry
LOD: Limit of detection
LOQ: Limit of quantitation
LPS: lipopolysaccharide
NCA: Non-compartmental analysis
T_max_: Time of maximum concentration
