# Supplementary material for: Pharmacokinetics and pharmacodynamics of intra-articular isoflupredone following administration to horses with lipopolysaccharide-induced synovitis
Source: BMC Vet Res. 2022 Dec 13;18:436. doi: 10.1186/s12917-022-03537-5 (PMC9746120; doi:10.1186/s12917-022-03537-5)
Supplement: Supplementary file 1 — Additional file 1. [file 12917_2022_3537_MOESM1_ESM.docx]

**Supplementary Material:**

**Type of models assessed:**

- 1 and 2 compartment models for both Csyn and Cp
- Unidirectional and bidirectional Kap
- Incorporation of a transduction (delay) compartment for Kap
- Effect of LPS on both Kap and CLsyn and CL
- Models without a CLsyn
- Models with and without random effects (etas) on the main structural parameters
- Additive, multiplicative and mixed residual error models for both analytes

Based on the estimated values of the parameters and their good precision, and based on an examination of the diagnostic plots, the model found to best and most adequately fit the data was a model with one compartment for both analytes, a Cl parameter for both analytes, a unidirectional Kap (Csyn to Cp) that was dependent on LPS, inclusion of random effects for all fixed effects in the model and a multiplicative error for both analytes.
